# Clinical value and molecular role of PRDXs family in prostate cancer

**DOI:** 10.3389/fonc.2025.1713670

**Published:** 2025-12-09

**Authors:** Yong Yao, Quan Zheng, Chen Qian, Peng Gu, Minhao Zhang, Zhou Zhang

**Affiliations:** 1 Department of Urology, Xishan People’s Hospital of Wuxi City, Kangda College of Nanjing Medical University, Wuxi, Jiangsu, China; 2 Department of Otorhinolaryngology, The Affiliated Suqian First People’s Hospital of Nanjing Medical University, Suqian, China; 3 Department of Clinical Laboratory, Xishan People’s Hospital of Wuxi City, Kangda College of Nanjing Medical University, Wuxi, Jiangsu, China; 4 Central Laboratory, Xishan People’s Hospital of Wuxi City, Kangda College of Nanjing Medical University, Wuxi, Jiangsu, China

**Keywords:** PRDXs, prostate cancer, expression, clinical value, molecular role

## Abstract

**Objectives:**

The purpose of this study is to explore the clinical value and molecular role of the peroxiredoxins (PRDXs) family in prostate cancer (PCa).

**Methods:**

We first analyzed the differentially expressed genes (DEGs) in Prostatic Adenocarcinoma (PRAD) using the Cancer Genome Atlas (TCGA) database, and then demonstrated the expression of six members of the PRDXs family in PRAD. Subsequently, we evaluated the expression of the PRDXs family using PCa cells and tissues. we also analyzed the diagnosis and overall survival (OS) of the PRDXs family in PCa. We used online tools to analyze the expression of PRDX4 in pan-cancer, the proteins interacting with it, as well as the amino acid regions and sites to pathogenicity. We used CCK8 and transwell assay to detect the proliferation and invasion of PCa cells after silencing PRDX4. Finally, we predicted traditional Chinese medicine drugs targeting PCa with PRDX4.

**Results:**

We found that PRDX2 and PRDX4 were highly expressed in PRAD through the TCGA database. Compared with prostate epithelial cells, PRDX2, PRDX3, PRDX4, and PRDX6 were expressed higher in PCa cells. In PCa tissues, the PRDXs family is widely expressed positively (P<0.05). The PRDXs family has relatively low diagnostic value in PCa, except for PRDX4. Based on the above results, we selected PRDX4 for molecular role detection. We found that the expression of PRDX4 in PCa was higher than that in more than half of the cancer types in pan-cancer. We found that there are eight proteins interacting with PRDX4. The pathogenic amino acid regions and sites of PRDX4 protein mutation that are prone to disease were mainly concentrated in the area after the 50th amino acid. We found that silencing PRDX4 slowed down the proliferation and invasion of PCa cells. Finally, we found that there are 14 traditional Chinese medicines targeting PCa with PRDX4, among which 5 have statistical differences, and Shi Liu Zi may be the best targeted traditional Chinese medicine drug.

**Conclusion:**

This study found that PRDX4 is highly expressed in PCa, which may promote the phenotypic progression of PCa cells and has high clinical value.

## 1 Introduction

Prostate cancer (PCa) is the second most common cancer in men worldwide (1). In recent years, its incidence rate has increased year by year around the world, posing a serious threat to men’s health (2, 3). Approximately 1.5 million new cases of PCa are diagnosed annually worldwide (1). Among them, prostatic adenocarcinoma (PRAD) accounts for over 95% of all prostate cancers (4). Management includes active surveillance, prostatectomy, or radiation therapy, depending on risk of progression (5). For patients with higher-risk disease, radiation therapy or radical prostatectomy are reasonable options (5, 6). Therefore, the discovery of diagnostic and prognostic monitoring markers for PCa remains crucial.

Peroxiredoxins (PRDXs) are a recently studied family of antioxidant proteins that play important roles in antioxidant defense and peroxide detoxification (7). The PRDXs family of mammals contains six members, namely PRDX1-6, which is a multifunctional protein family involved in cell growth, differentiation, and apoptosis (8). Earlier studies showed that PRDX proteins possess the ability to regulate the proliferation, metabolism and immune regulation of tumor cells, along with their involvement in the pathological regulation or protection of various types of cancer (9), including head and neck squamous cell carcinoma (10), colorectal cancer (11), hepatocellular carcinoma (12–14), breast cancer (15), Non-small-cell lung cancer (16) and so on. Our previous studies have also shown that the PRDXs family has varying degrees of expression and corresponding roles in oral cancer (17), liver cancer (18), colorectal cancer (19), lung cancer (20), and so on. However, there is limited research on members of the PRDXs family in PCa. Some studies suggested that PRDX3 and PRDX4 was overexpressed in PCa of cancer microarray datasets (21, 22). However, there is currently no systematic analysis of the expression of PRDXs family members in PCa tissues and cells, as well as their clinical value and molecular role in PCa.

This study aims to comprehensively utilize the TCGA database, PCa cells, and PCa tissues to analyze the expression and clinical value of the PRDXs family in PCa, and further explore its molecular role in PCa, opening up new ideas for the clinical diagnosis and treatment of PCa.

## 2 Materials and methods

### 2.1 Bioinformatics analysis of PRAD

The volcano plot and heat map were created using TCGA databases to illustrate the distribution of DEGs in PRAD. The expression of PRDXs in PRAD was depicted using the TCGA databases, and a statistical difference analysis was conducted. The diagnostic value of the PRDXs family in PRAD was obtained by drawing the ROC curve based on the clinical data in the TCGA database. The data analysis of this part is all based on R software (version 4.02).

### 2.2 Clinical information of patients with PCa

This study mainly relies on the clinical data of PCa patients in the TCGA database. According to the screening rules, 484 case data of PCa patients were obtained (**Table 1**). Among them, 217 cases are under or equal to 60 years old, and 267 cases are over 60 years old; In terms of T staging, the number of patients belonging to T2, T3 and T4 stages was 180, 287 and 10 respectively. Additionally, there were 7 PCa patients whose stage was unknown; In terms of N staging, the number of patients belonging to N0 and N1 stages was 338 and 78 respectively, and there were also 68 PCa patients whose stage was unknown; In terms of survival time, there are 399 PCa patients with a survival time of no more than 5 years and 85 patients with a survival time of more than 5 years; In terms of survival ending, there were 474 PCa patients alive and 10 PCa patients dead.

**Table 1:** Clinical information of PRAD patients from TCGA database.

Clinical features	Number	Percentage (%)
Age (Years)		
≤60	217	44.83
>60	267	55.17
T		
T1	0	0
T2	180	37.19
T3	287	59.30
T4	10	2.07
Not Known	7	1.45
N		
N0	338	69.83
N1	78	16.12
Not Known	68	14.05
M		
Not Known	484	100
Stage		
Not Known	484	100
Survival Time (Years)		
≤5	399	82.44
>5	85	17.56
Survival Ending		
Alive	474	97.93
Dead	10	2.07
All	484	100

### 2.3 Immunohistochemical staining

All the primary antibodies are from Wuhan Sanying Biotechnology Co., LTD. Among them, the product number of PRDX1 antibody is 15816-1-AP, that of PRDX2 antibody is 10545-2-AP, that of PRDX3 antibody is 10664-1-AP, that of PRDX4 antibody is 10703-1-AP, and that of PRDX5 antibody is 17724-1-AP. The product number of PRDX6 antibody is 13585-1-AP. All antibodies are hosted by rabbits and can be used for IHC. The dilution ratio is all 1:500. The secondary antibody was purchased from Abcam Company with the item number ab205718, and the dilution ratio is 1:2000. Fresh prostate cancer tissues should be quickly frozen in liquid nitrogen or dry ice, and then stored in a -80°C refrigerator for future use. The tissue blocks were cut into sections 6um thick using a cryostat. Fix the sections in the fixative (methanol) for 5 to 10 minutes. Incubate the sections with blocking solution (BSA) at 37°C for 30 minutes to reduce non-specific binding. Add the primary antibody and incubate at room temperature for 1–2 hours. Wash the sections with PBS buffer to remove unbound primary antibodies. Add the secondary antibody and incubate at room temperature for 30 minutes. Wash the sections again with PBS buffer to remove unbound secondary antibodies. Subsequently, the sections were stained with 0.01% 3,3 ‘-diaminobenzidine at room temperature for 5 minutes, and then rinsed three times in PBS at room temperature (each time for 3 minutes). After counterstaining with hematoxylin at room temperature for 3 minutes, rinse three times in PBS at room temperature (each time for 3 minutes). All samples were imaged using the CX31 optical microscope (Olympus).

### 2.4 Cell culture and transfection

The Prolate epithelial cell (RWPE-1) and PCa cell (22RV1) were both purchased from American Type Culture Collection (ATCC) company and identified by STR. RWPE-1 and 22RV1 cells in the logarithmic growth phase and in good growth condition were seeded in 6-well cell culture plates at a ratio of 2×10^5^ cells per well and incubated overnight in a 37°C, 5% CO2 incubator. Two hours before transfection, switch to serum-free medium. Dilute 10ul of siRNA (20uM) with 200ul of serum-free medium and gently mix with a pipette tip. Then, add 5ul of Lipofectamine 2000, gently mix well, and let it stand at room temperature for 20 minutes. Add 215uL of the mixed solution to each culture well respectively, and gently shake the cell culture plate back and forth to mix the mixed solution evenly with the culture medium in the culture plate. The cells were placed in a 37°C, 5% CO2 incubator for 6 hours and then replaced with normal medium. The sense of siRNA-1 is GCUGUGAUCGAUGGAGAAUUUTT(5’-3’) and the antisense of siRNA-1 is AAAUUCUCCAUCGAUCACAGCTT (5’-3’); The sense of siRNA-2 is GCUCUGUUGAUUCACAGUUUATT(5’-3’) and the antisense of siRNA-2 is UAAACUGUGAAUCAACAGAGCTT (5’-3’); The sense of siRNA-3 is GCUGAAGUAUUUCGAUAAACUTT(5’-3’) and the antisense of siRNA-3 is AGUUUAUCGAAAUACUUCAGCTT (5’-3’).

### 2.5 Western blot

All the primary antibodies are from Wuhan Sanying Biotechnology Co., LTD. Among them, the product number of PRDX1 antibody is 15816-1-AP, that of PRDX2 antibody is 10545-2-AP, that of PRDX3 antibody is 10664-1-AP, that of PRDX4 antibody is 10703-1-AP, and that of PRDX5 antibody is 17724-1-AP. The product number of PRDX6 antibody is 13585-1-AP. The product number of GPAPDH antibody is 10494-1-AP. All antibodies are hosted by rabbits and can be used for Western blot. Treated cells were harvested and lysed in RIPA buffering solution, and were determined using BCA. Protein samples (30ug/lane) from each group were separated using 10% SDS−PAGE, transferred onto PVDF membranes and blocked in BSA. Next, the membranes were immersed in primary antibody at 4˚C overnight. Then they were rinsed with TBST (0.1% Tween−20, cat. no. T1085, Solarbio) three times for 10 min each. Incubation with goat Anti−Rabbit IgG H&L (HRP, SA00001-4; Proteintech Group, Inc, 1:5,000) was performed at room temperature for1 h, followed by rinsing with TBST three times for 10 min each. Protein detection was performed using BeyoECL Star (cat. no. P0018AS, beyotime). The bands were quantified using the Image Lab System version 6.1.

### 2.6 Reverse transcription-quantitative PCR

Total RNA was extracted from eight cell lines using TRIzol reagent (Beyotime, R0016). Using BeyoRT ™ The II cDNA first strand synthesis kit (Beyotime, D7168M) synthesizes complementary DNA (cDNA). BeyoFast ™ SYBR Green qPCR Mix (Beyotime, D7265) and CFX 96 thermal circulator (Bio Rad) were used for RT-qPCR. Perform RNA extraction, cDNA synthesis and qPCR according to the instructions. RT-qPCR was carried out using the following conditions: preheating for 2 min at 95 °C; and then repeating 40 cycles in 95 °C for 15 sec and 56 °C for 30 sec. The primer sequences of PRDX4 were as follows: Forward, 5’- CTCCCTGCACCTAAGCAAAG -3’and Reverse, 5’- CTGTCGCCAAAAGCGATAAT -3’. Using GAPDH as an internal reference gene and the primer sequence were as follows: Forward, 5’- TCAAGAAGGTGGTGAAGCAGG-3’ and Reverse,5’-TCAAAGGTGGAGGAGTGGGT -3’. The relative expression level of PRDX4 in this study was calculated using the 2^−ΔΔCq^ formula. Repeat the experiment three times.

### 2.7 Cell counting kit-8

Firstly, 22RV1 cells in logarithmic growth phase with good growth status were seeded at 5×10^3^ cells/well into a 96 well cell culture plate, and then cultured overnight in a 37°C, 5% CO_2_ incubator. Two hours before transfection, replace the culture medium with serum-free 22RV1 cell specific culture medium. For each transfection sample, prepare according to the following method: mix gently with 0.5uL Lipofectamine 2000, 0.5 uL siRNA (20 u M), and 19uL culture medium, and let it stand at room temperature for 20 minutes. Add 20 uL of mixed solution to each culture well, gently shake the cell culture plate back and forth to mix the mixed solution with the culture medium in the plate. Then, the cells were cultured in CO_2_ at 37°C for 6 hours and replaced with normal medium. After replacing the normal culture medium, continue to culture for 24 hours and 48 hours, and perform CCK8 detection on the cells respectively. After the required time for cell culture, 10 uL of CCK8 was added to each well and incubated at 37°C for 2 hours. Then, the absorbance values of each well were measured using an enzyme-linked immunosorbent assay OD 450.

### 2.8 Transwell experiment

Inoculate 22RV1 cells, which are in logarithmic growth phase and in good growth condition, into a 6-well cell culture plate at 2 × 10^5^ cells/well, and then culture overnight in a 37°C, 5% CO2 incubator. Two hours before transfection, replace the serum-free culture medium. For each transfection sample, prepare according to the following method: mix gently with 10 uL Lipofectamine 2000, 10 uL siRNA (20 u M), and 180uL culture medium, and let it stand at room temperature for 20 minutes. Add 200 uL of mixed solution to each culture well, gently shake the cell culture plate back and forth to mix the mixed solution with the culture medium in the plate. Then, the cells were cultured in CO2 at 37°C for 6 hours and replaced with normal medium. Use trypsin to digest and collect cells separately, centrifuge at 1200 rpm for 5 minutes, and wash twice with PBS to remove residual serum. Dilute the cell concentration to 3 × 10^5^/ml. Melt Matrigel at 4°C one day in advance, and then dilute Matrigel with serum-free medium to a final concentration of 1mg/ml. Add 800 uL of culture medium containing 10% FBS pre cooled at 4°C to a 24 well plate, and place it in a transwell chamber. Vertically add 100 uL of Matrigel with a final concentration of 1mg/ml in the center of the bottom of the transwell chamber, and incubate at 37°C for 1 hour. After Matrigel incubation, add 200 uL of each group of cell suspensions to the transwell chamber and incubate in a 5% CO_2_ incubator at 37°C. After replacing the normal culture medium, continue to culture for 24 hours and 48 hours, and the cells were tested separately. Take out the Transwell chamber, carefully clean the chamber with PBS, fix the cells with 70% ice ethanol solution for 1 hour. Stain with 0.5% crystal violet staining solution, leave at room temperature for 20 minutes, wash with PBS once, wipe off the non-invasion cells on the upper chamber side, observe and take photos under a microscope.

### 2.9 The utilization of online websites and tools

The Overall Survival of the PRDXs family in prostate cancer was determined using the Gene Expression Profiling Interactive Analysis 2(GEPIA2) online tool. The expression patterns of PRDX4 across pan-cancer tissues were assessed using the University of Alabama at Birmingham Cancer Data Analysis Portal (UALCAN). The Universal Protein (Uniprot) online website has revealed proteins that may interact with PRDX4. AlphaFold Protein Structure Database online website has revealed the heat map of pathogenicity of PRDX4 protein mutation and the three-dimensional structure diagram of the pathogenicity of PRDX4 protein mutation.

### 2.10 Statistical analysis

Statistical analysis was performed using SPSS 22.0 and GraphPad Prism 7.0. The results obtained were presented as mean ± standard deviation (x̄ ± s), and significant differences between the two groups were assessed with the Student’s t-test. Counting data is represented by examples, and a chi-square test was implemented for comparing two kinds of groups. P<0.05 was considered statistically significant.

## 3 Result

### 3.1 Differential expression genes of PRAD in TCGA database

**Figure 1:**
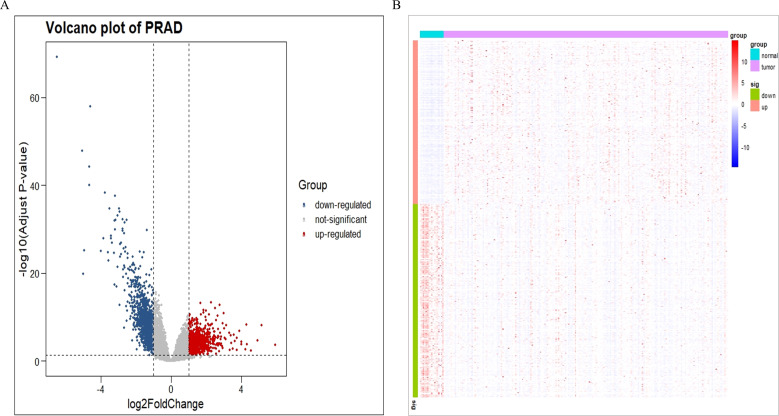
Differential expression genes of PRAD in TCGA database. **(A)** A volcano plot of PRAD. **(B)** A heat map of PRAD.

We use R language to analyze DEGs in PRAD. The results are presented in the form of volcano (**Figure 1A**) and heat maps (**Figure 1B**). We found that 1160 DEGs were down regulated (blue dots in the volcano plot), 984 DEGs were up regulated (red dots in the volcano plot), and 14716 non DEGs (gray dots in the volcano plot) in PRAD.

### 3.2 Expression of PRDXs family in PRAD in TCGA database

**Figure 2:**
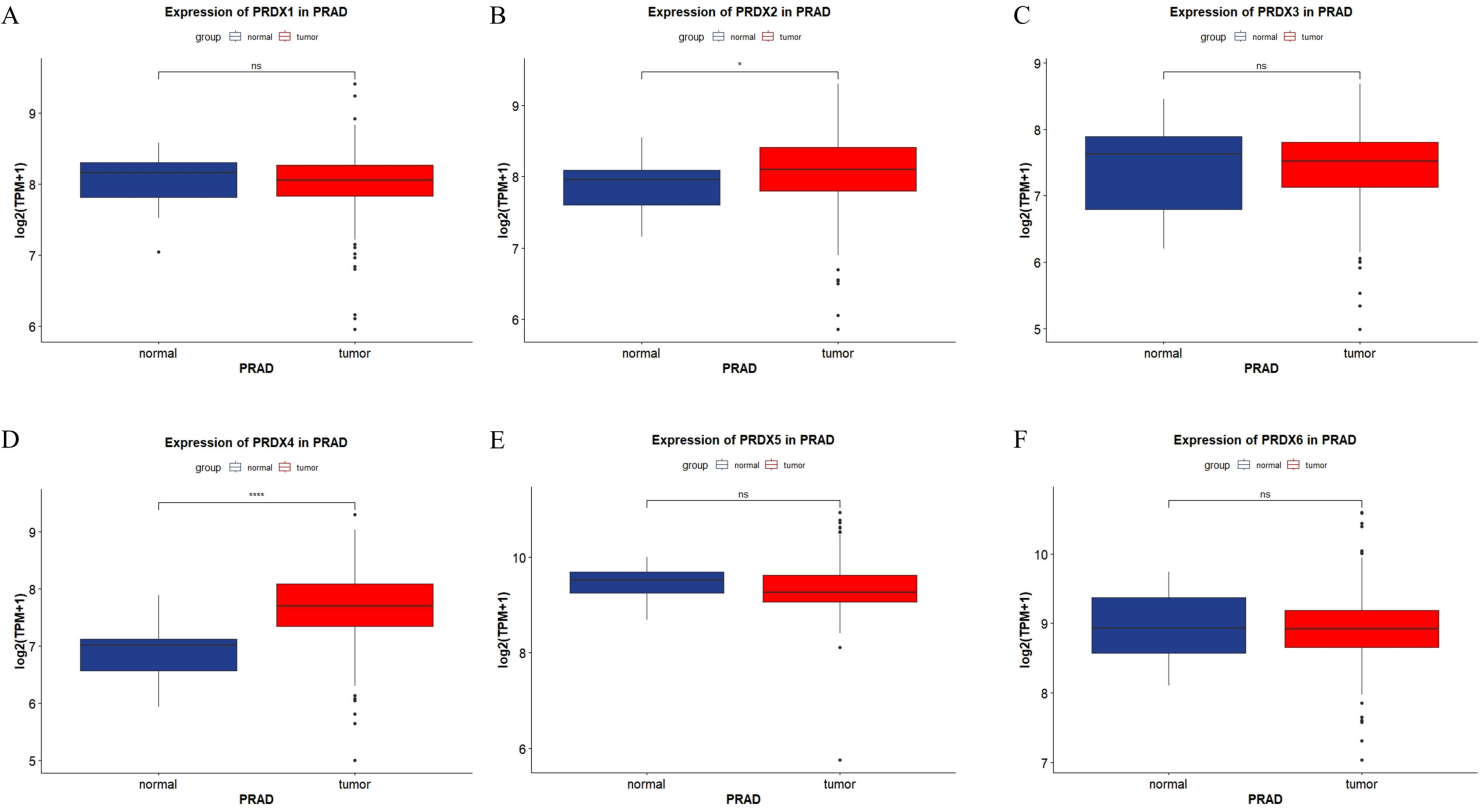
Expression of PRDXs family in PRAD in TCGA database. **(A)** Expression of PRDX1 in PRAD. **(B)** Expression of PRDX2 in PRAD. **(C)** Expression of PRDX3 in PRAD. **(D)** Expression of PRDX4 in PRAD. **(E)** Expression of PRDX5 in PRAD. **(F)** Expression of PRDX6 in PRAD. *P<0.05, ****P<0.0001.

We presented the expression of the PRDXs family in PRAD through the form of a box plot (**Figure 2**). The expression of PRDX2 was elevated in PRAD, and the difference was statistically significant (P<0.05) (**Figure 2B**). The expression of PRDX4 was elevated in PRAD, and the difference was statistically significant (P<0.0001) (**Figure 2D**). However, other members of the PRDXs family, including PRDX1, PRDX3, PRDX5, and PRDX6, showed no statistically significant difference in expression in PRAD (P>0.05) (**Figures 2A, C, E, F**).

### 3.3 Expression of PRDXs family in RWPE-1 and 22RV-1 cells

**Figure 3:**
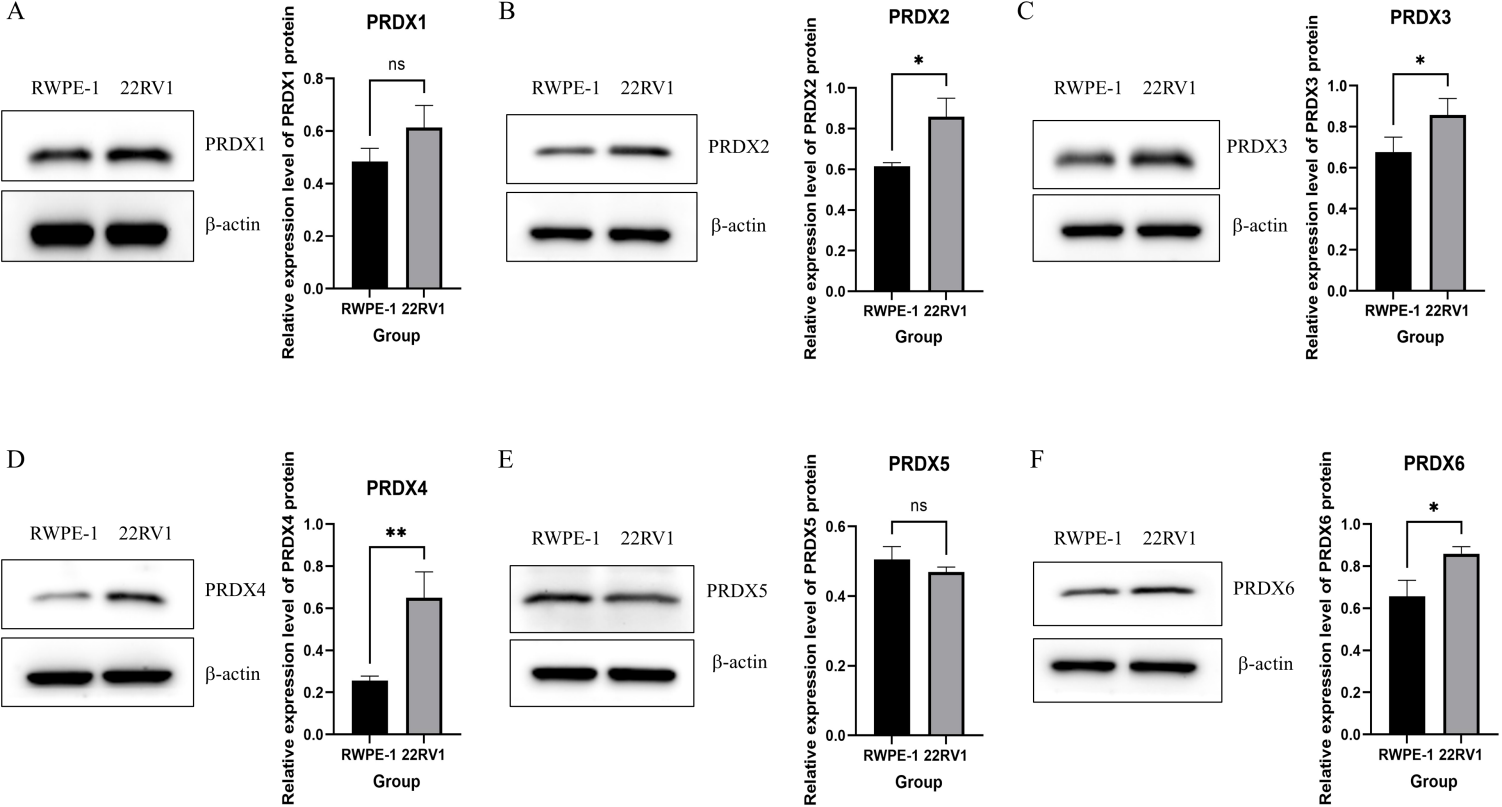
Expression of PRDXs family in RWPE-1 and 22RV-1 cells. **(A)** Expression of PRDX1 in RWPE-1 and 22RV-1 cells. **(B)** Expression of PRDX2 in RWPE-1 and 22RV-1 cells. **(C)** Expression of PRDX3 in RWPE-1 and 22RV-1 cells. **(D)** Expression of PRDX4 in RWPE-1 and 22RV-1 cells. **(E)** Expression of PRDX5 in RWPE-1 and 22RV-1 cells. **(F)** Expression of PRDX6 in RWPE-1 and 22RV-1 cells. *P<0.05, **P<0.01. All experimental biological and technical replicates were conducted at least three times.

We used Western blot technology to detect the expression of PRDXs family in prostate epithelial cells (RWPE-1) and PCa cells (22RV1) (**Figure 3**). The expression of PRDX2, PRDX3 and PRDX6 in PCa cells (22RV1) is higher than that in prostate epithelial cells (RWPE-1), and the difference is statistically significant(P<0.05) (**Figures 3B, C, F**). The expression of PRDX4 in PCa cells (22RV1) is higher than that in prostate epithelial cells (RWPE-1), and the difference is statistically significant(P<0.01) (**Figure 3D**). However, there was no statistically significant difference in the expression of other members of the PRDXs family, including PRDX1 and PRDX5, between prostate cancer cells and prostate epithelial cells(P>0.05) (**Figures 3A, E**).

### 3.4 Positive proportion of PRDXs family in normal prostate and PCa tissues

**Figure 4:**
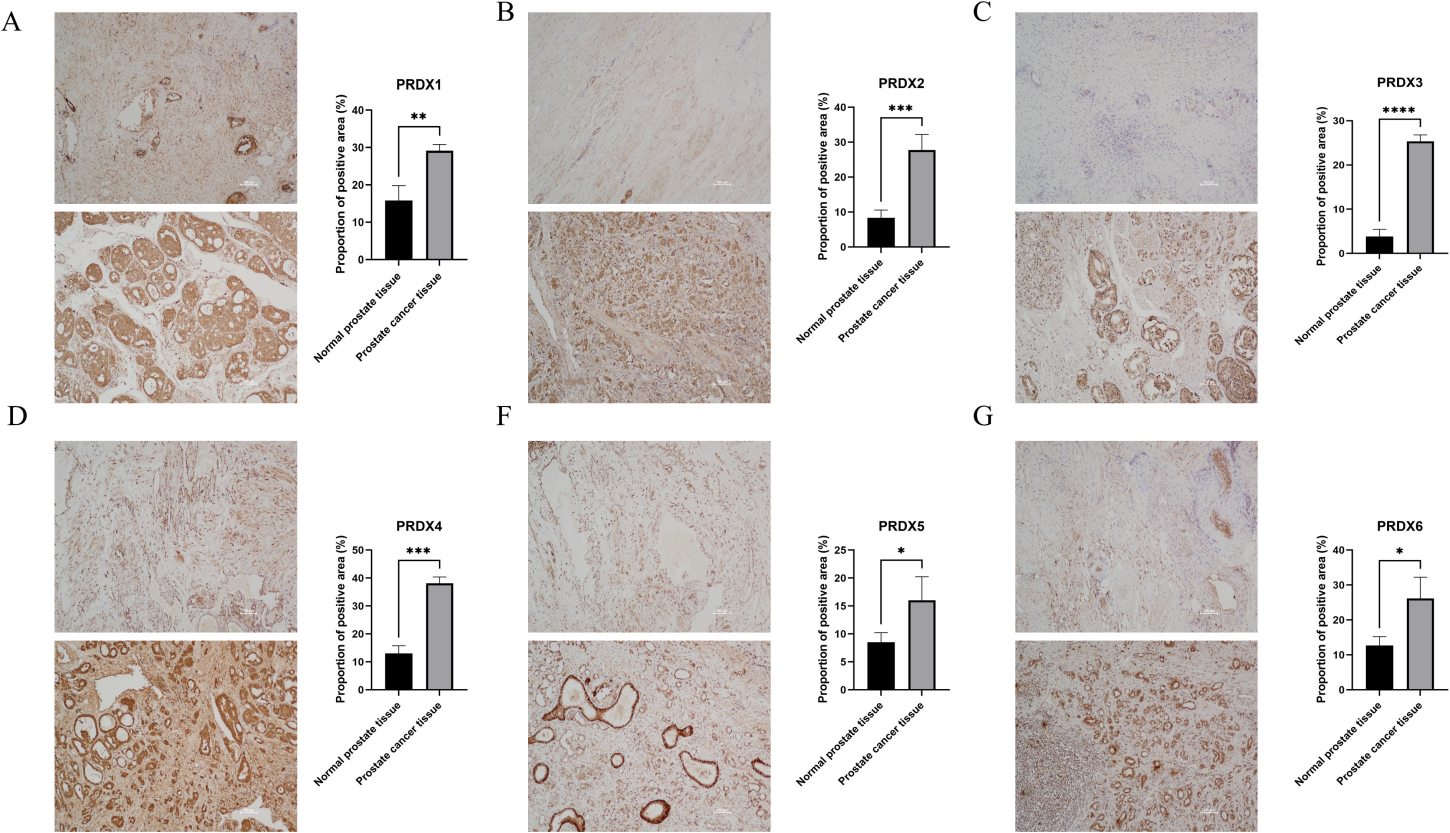
Positive proportion of PRDXs family in normal prostate and PCa tissues. **(A)** Positive proportion of PRDX1 in normal prostate and PCa tissues. **(B)** Positive proportion of PRDX2 in normal prostate and PCa tissues. **(C)** Positive proportion of PRDX3 in normal prostate and PCa tissues. **(D)** Positive proportion of PRDX4 in normal prostate and PCa tissues. **(E)** Positive proportion of PRDX5 in normal prostate and PCa tissues. **(F)** Positive proportion of PRDX6 in normal prostate and PCa tissues. *P<0.05, **P<0.01. ***P<0.001,****P<0.0001. All experimental biological and technical replicates were conducted at least three times.

In PCa tissues, the PRDXs family is widely expressed positively (P<0.05) (**Figure 4**). Specifically, compared with normal prostate tissue, PRDX3 showed the most significant positive expression in PCa tissue(P<0.0001) (**Figure 4C**); In addition, there is also a significant difference in the positive expression of PRDX2 and PRDX4 in PCa tissues(P<0.001) (**Figure 4B, D**); PRDX1 followed closely behind once again(P<0.01) (**Figure 4A**); Finally, the positive expression of PRDX5 and PRDX6 was least significant in prostate cancer tissues(P<0.05) (**Figures 4E, F**).

### 3.5 Diagnostic value of PRDXs family in PCa

**Figure 5:**
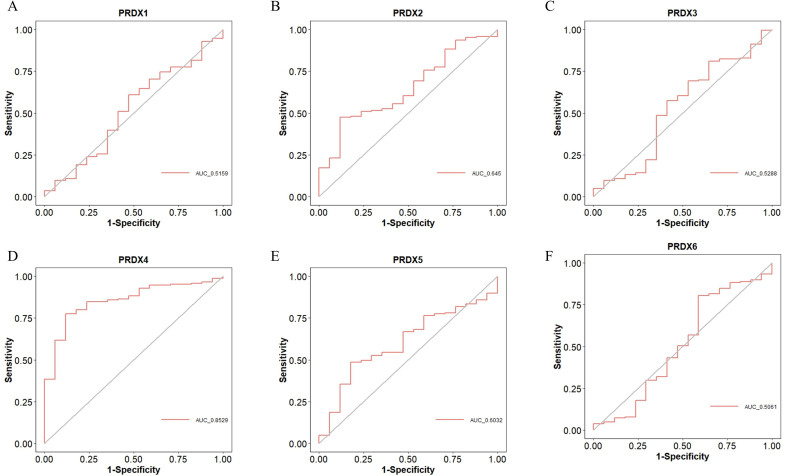
Diagnostic value of PRDXs family in PCa. **(A)** Diagnostic value of PRDX1 in PCa. **(B)** Diagnostic value of PRDX2 in PCa. **(C)** Diagnostic value of PRDX3 in PCa. **(D)** Diagnostic value of PRDX4 in PCa. **(E)** Diagnostic value of PRDX5 in PCa. **(F)** Diagnostic value of PRDX6 in PCa.

We used Receiver Operating Characteristic (ROC) curves to analyze the diagnostic value of PRDXs family for PCa (**Figure 5**). PRDX4 has a high diagnostic value for PCa, with an Area Under the Curve (AUC) of 0.8529 and a cut-off value of 7.308, corresponding to coordinates (0.882, 0.775) (**Figure 5D**). Other members of the PRDXs family, including PRDX1, PRDX2, PRDX3, PRDX5, and PRDX6, have low diagnostic value for PCa (all AUC<0.7). Specifically, the AUC of PRDX1 for PCa is 0.5159, with a cut-off value of 8.161 and corresponding coordinates of (0.529, 0.608) (**Figure 5A**); the AUC of PRDX2 for PCa is 0.645, with a cut-off value of 8.163 and corresponding coordinates of (0.882, 0.475) (**Figure 5B**); the AUC of PRDX3 for PCa is 0.5288, with a cut-off value of 7.889 and corresponding coordinates of (0.353, 0.809) (**Figure 5C**); the AUC of PRDX5 for PCa is 0.6032, with a cut-off value of 9.243 and corresponding coordinates of (0.824, 0.485) (**Figure 5E**); the AUC of PRDX6 for PCa is 0.5061, with a cut-off value of 9.265 and corresponding coordinates of (0.412, 0.804) (**Figure 5F**).

### 3.6 Overall survival of PCa with different expression levels of PRDXs family

**Figure 6:**
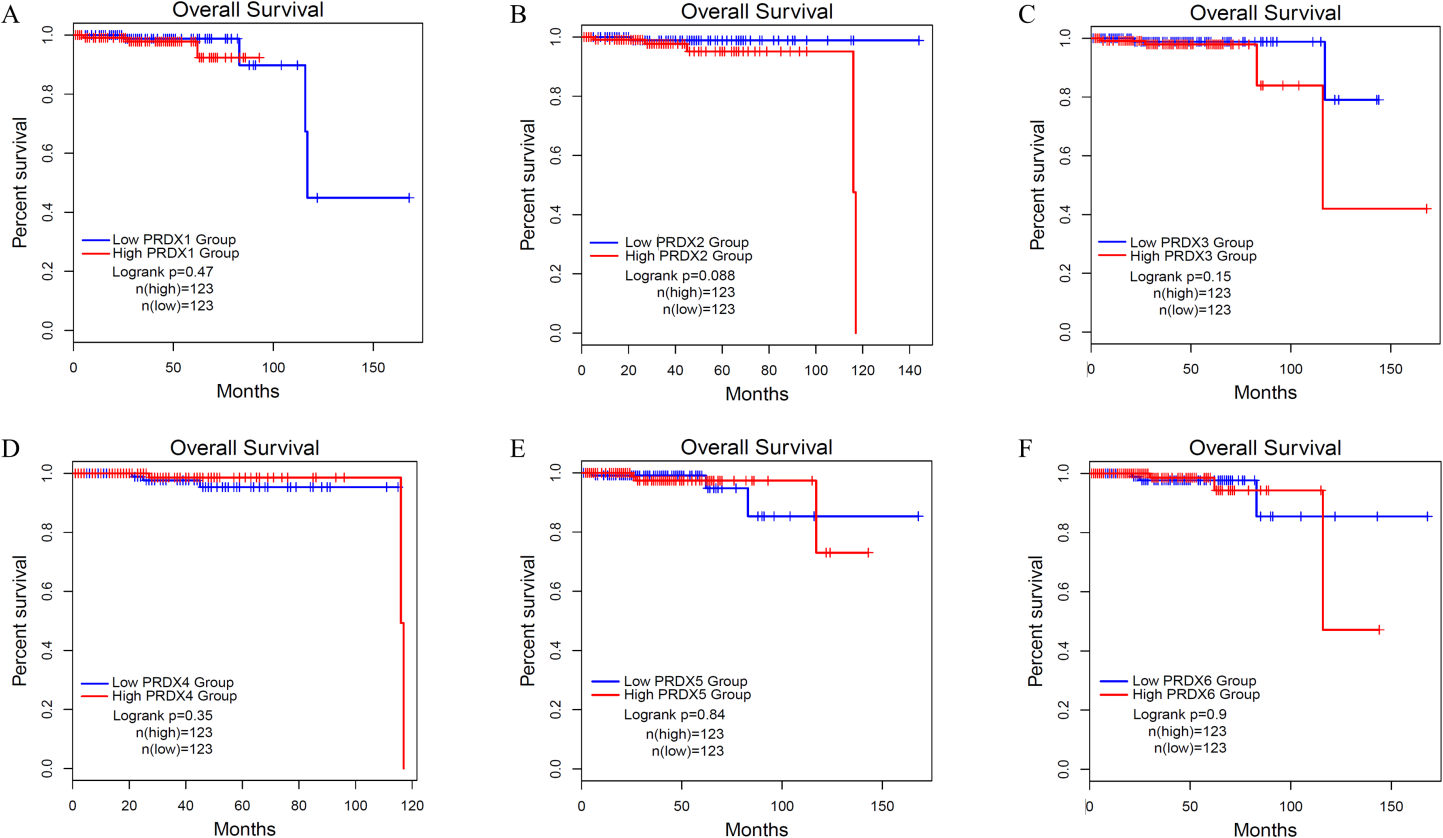
Overall survival (OS) of PCa with different expression levels of PRDXs family. **(A)** OS of PCa with different expression levels of PRDX1. **(B)** OS of PCa with different expression levels of PRDX2. **(C)** OS of PCa with different expression levels of PRDX3. **(D)** OS of PCa with different expression levels of PRDX4. **(E)** OS of PCa with different expression levels of PRDX5. **(F)** OS of PCa with different expression levels of PRDX6.

We used Kaplan Meier (KM) survival curves to analyze the OS of PCa patients with different expression of PRDXs family (**Figure 6**). Unfortunately, all members of the PRDXs family cannot be used as prognostic monitoring indicators for OS in PCa patients, as their KM curves do not have statistical differences(P>0.05).

### 3.7 Expression of PRDX4 in pan-cancer of TCGA database, its interaction protein and pathogenicity

**Figure 7:**
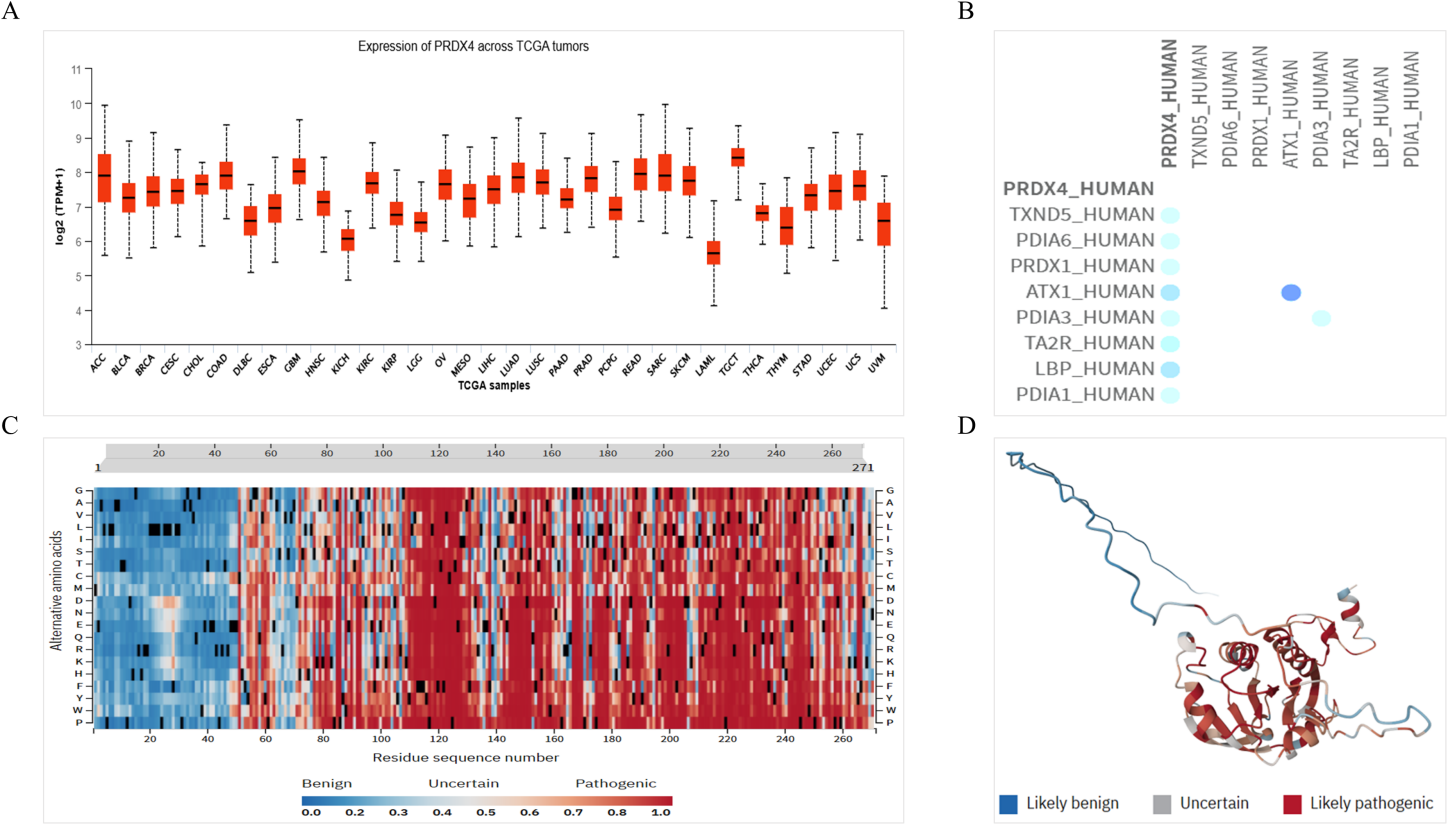
Expression of PRDX4 in pan-cancer of TCGA database, its interaction protein and pathogenicity. **(A)** Expression of PRDX4 in pan-cancer of TCGA database. **(B)** Proteins interacting with PRDX4. **(C)** A heat map of pathogenicity of PRDX4 protein mutation. **(D)** A three-dimensional structure diagram of the pathogenicity of PRDX4 protein mutation.

Based on the above results, we believe that PRDX4 has significant clinical value in PCa, and therefore we will continue to explore the molecular role of PRDX4 in the PCa. Firstly, we analyzed the expression of PRDX4 in pan-cancer of TCGA database. It was found that the expression of PRDX4 in the prostate ranks among the top in pan cancer, accounting for over half of pan cancer (**Figure 7A**). We continue to analyze the proteins that may interact with PRDX4, laying the foundation for exploring the molecular mechanisms of PRDX4 in prostate cancer in the future. We found 8 proteins that may interact with PRDX4, including TXND5, PDIA6, PRDX1, ATX3, PDIA3, TA2R, LBP, and PDIA1(**Figure 7B**). This suggests that PRDX4 may be more closely related to members of the PDIA family. We proceeded to analyze the pathogenic amino acid regions and sites of PRDX4 protein mutation. The results showed that the pathogenic amino acid regions and sites of PRDX4 protein mutation that are prone to disease were mainly concentrated in the area after the 50th amino acid (red region), especially between the 110-130th, 145-155th, and 195-255th regions, which were more severe (**Figures 7C, D**).

### 3.8 Effect of PRDX4 on the phenotype of 22RV-1 cells

**Figure 8:**
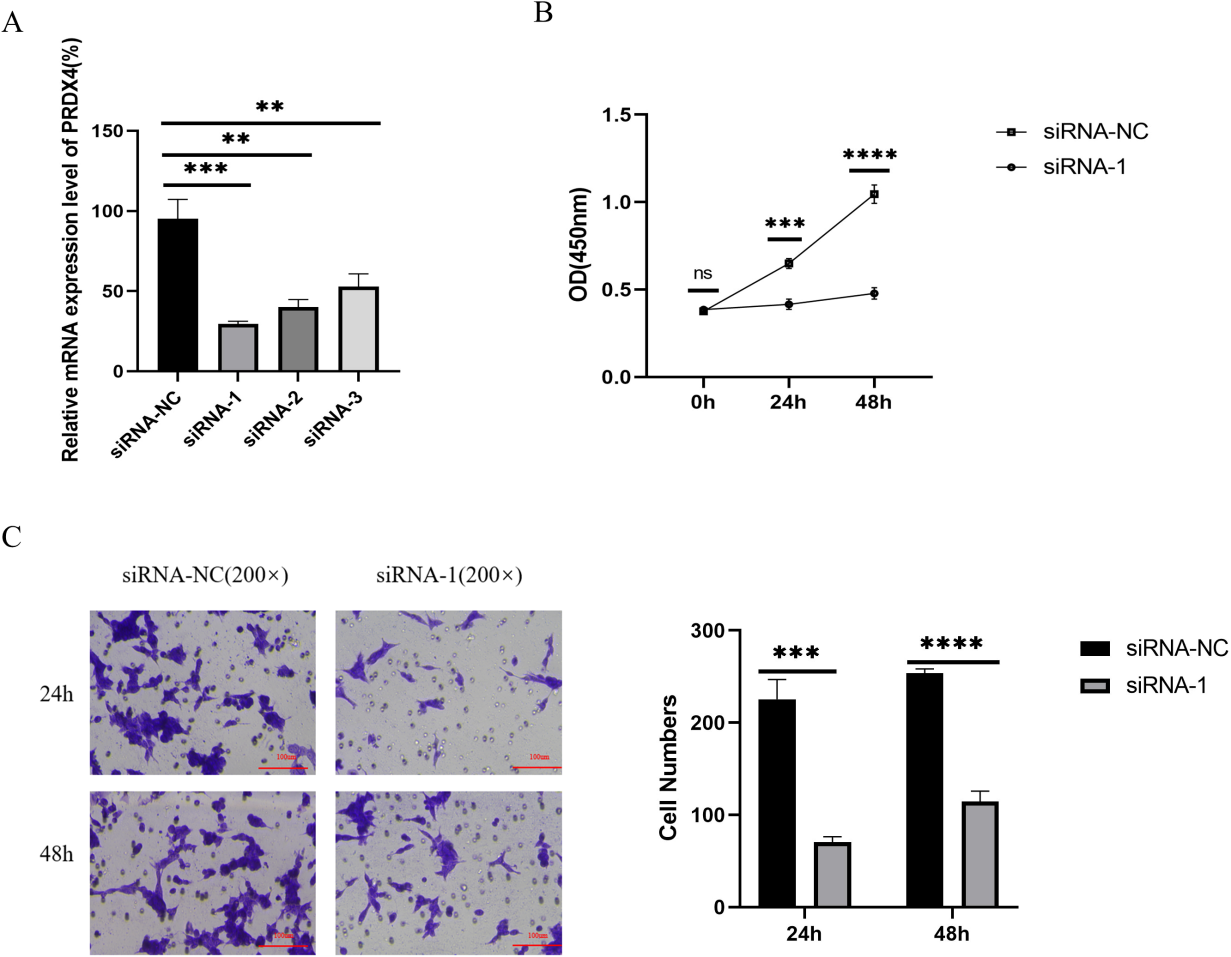
Effect of PRDX4 on the phenotype of 22RV-1 cells. **(A)** Verification of the silencing efficiency of siRNA-PRDX4 in 22RV-1 cells. **(B)** Effect of silencing PRDX4 on the proliferation of 22RV-1 cells. **(C)** Effect of silencing PRDX4 on the invasion of 22RV-1 cells. All experimental biological and technical replicates were conducted at least three times. **P<0.01. ***P<0.001,****P<0.0001

We silenced PRDX4 in prostate cancer (22RV-1) cells using siRNA. The results showed that the silencing efficiency of the first siRNA-PRDX4(siRNA-1) was the highest (**Figure 8A**). Immediately after, we examined the proliferation and invasion of 22RV-1 cells silenced with PRDX4. The results showed that compared with the control group (siRNA-NC), the cell proliferation and invasion in the silencing group (siRNA-1) were accelerated, and the differences were statistically significant (all P<0.05) (**Figures 8B, C**).

### 3.9 Prediction of traditional Chinese medicine targeting PCa with PRDX4

For future clinical translation, we used Coremine Medical tool to preliminarily explore traditional Chinese medicine targeting PCa with PRDX4, laying the foundation for subsequent experimental validation. The results showed that there were 14 traditional Chinese medicines targeting PCa with PRDX4, among which 5 had statistical differences, including Shi Liu Zi, Yuan Can E, Can Sha, Shi Liu Hua, and Shi Liu Pi (all P<0.05). In the future, further experiments are expected to confirm that Shiliuzi may be the most promising traditional Chinese medicine targeting PCa with PRDX4 (**Supplementary Table 1**).

## 4 Discussion

PCa, as one of the common cancers in men, seriously endangers the health of men worldwide (23). In recent years, although the overall prognosis of PCa has been good, the prognosis for patients with advanced stage and metastasis remains poor (3, 24). Therefore, seeking diagnostic and prognostic monitoring biomarkers for PCa and exploring their molecular roles in PCa remains important and urgent.

Previous studies have shown a certain correlation between the PRDXs family and the occurrence and development of PCa (21, 22, 25–29). Similar to this study, Basu A et al. and Ummanni R et al. found that all PRDXs exhibited elevated protein expression in PCa cell lines, compared with non-tumor cells (22, 30). However, this study found that PRDX2, PRDX3, PRDX4 and PRDX6 were also upregulated in PCa cells, which may be due to the inconsistent use of prostate cell lines. In PCa tissues, this study found the PRDXs family is widely expressed positively, with PRDX2, PRDX3, and PRDX4 showing the most significant differences, and this is basically consistent with their research results (22, 30). Similarly, Priyanka Balasubramanian et al. found that PRDX2 is overexpressed in various cancers, including PCa, which is consistent with the findings of this study (31). Meanwhile, using the TCGA database, this study found that PRDX2 and PRDX4 were highly expressed in PCa, while there was no difference in expression among other members of the PRDXs family. In terms of clinical value, only PRDX4 in the PRDXs family has significant diagnostic value for PCa. At present, Prostate Specific Antigen (PSA) is still the core tumor marker used for diagnosing PCa in clinical practice, combined with Prostatic Acid Phosphatase (PAP) and Prostate Cancer Antigen 3(PCA3) for joint evaluation. Multiple studies have shown that PSA has a high diagnostic value for prostate cancer, with its AUC exceeding 70% (32, 33). Specifically, PSA has high sensitivity and poor specificity in diagnosing prostate cancer (34). In addition, PAP and PCA3 have gradually been applied in clinical diagnosis of PCa in recent years, but they are not yet mature (35). This study found that PRDX4 has a high diagnostic value for PCa, with an AUC of 0.8529. In the future, if PRDX4 is combined with PSA, PAP, and PCA3 for the diagnosis of prostate cancer, it may greatly improve the current limitation of poor PSA specificity and further enhance the sensitivity of diagnosing PCa. However, all members of the PRDXs family have no significant clinical value for the prognostic monitoring of the PCa, and this may be influenced by short follow-up times or sample bias. In the future, we will try to increase the follow-up time and sample size as much as possible to make this part of the results more objective. Anamika Basu et al. found that PRDX3 has prognostic monitoring value for Caucasian prostate cancer patients, but no prognostic monitoring value for African-American prostate cancer patients (22). This difference in outcome might be caused by the different stratification of prostate cancer patients. Based on the above, we have basically determined that PRDX4 is highly expressed in PCa and may have significant diagnostic value for PCa. Immediately after, we analyzed the expression of PRDX4 in pan-cancer. We found that the expression of PRDX4 in PCa was higher than that in more than half of the cancer types in pan-cancer. Given the high expression of PRDX4 in PCa, we believe that PRDX4 may function as an oncogene. Then we analyzed the proteins that might interact with PRDX4, which included TXND5, PDIA6, PRDX1, ATX3, PDIA3, TA2R, LBP, and PDIA1. It seems that PRDX4 may be more closely related to members of the PDIA family. Then, we analyzed the pathogenic amino acid regions and sites of PRDX4 protein mutation. We found that the pathogenic amino acid regions and sites of PRDX4 protein mutation that are prone to disease were mainly concentrated in the area after the 50th amino acid, especially in the region after the 110th amino acid, and most of them are located in the last four-fifths of the entire PRDX4 protein domain. Subsequently, we silenced PRDX4 in PCa cells using siRNA to clarify the effect of PRDX4 on the phenotype of PCa cells. We found that silencing PRDX4 could promote the proliferation and invasion of PCa cells. Currently, there is limited research on PRDX4 in PCa, but it has been extensively studied in some other cancers. Sun Yi Park et al. found that PRDX4 not only promotes gastric cancer cell invasion, but also promotes EMT migration (36). A study has found that there is an interaction between PRDX4 and TXNDC5 protein molecules in gastric cancer, and PRDX4 may reduce lymphocyte infiltration, affect the expression of multiple immune checkpoints, leading to anti-tumor immune suppression (37). Exploring the impact of PRDX4 on other phenotypes of prostate cancer cells could be one of our research directions in the future. For esophageal cancer, Mingming Tang et al. found the importance of the THUMPD3-AS1/miR-29a-3p/ELK1/PRDX4 axis as a key regulatory pathway in esophageal cancer, revealing its carcinogenic role in enhancing tumor invasiveness (38). TNF related apoptosis inducing ligand (TRAIL) is a promising target for developing anti-cancer therapies. Hua Qin Wang et al. demonstrated that TRAIL inhibits the PRDX4 gene at the transcriptional level, thereby promoting TRAIL induced cell death (39). Further investigation revealed that the β-catenin downstream gene ID2 is responsible for the oncogenic activity of PRDX4 in hepatocellular carcinoma (HCC) cells, promoting anchorage-independent growth and anoikis resistance (40). This seems to indicate that PRDX4 acts more as a downstream target in the process of tumor progression, directly affecting tumor progression. Therefore, further exploration of the upstream regulatory links of PRDX4 affecting the progression of prostate cancer seems to be what we should do in the future. For future clinical translation, we used Coremine Medical tool to preliminarily explore traditional Chinese medicine targeting PCa with PRDX4, laying the foundation for subsequent experimental validation. We found that there are five kinds of traditional Chinese medicine, including Shi Liu Zi, Yuan Can E, Can Sha, Shi Liu Hua, and Shi Liu Pi. In the future, further experiments are expected to confirm that Shiliuzi may be the most promising traditional Chinese medicine targeting PCa with PRDX4.

However, there are still some limitations to this study. The clinical value of PRDX4 in prostate cancer in this study depends on patient cases in the TCGA public database, and actual patient cases in clinical practice were not collected. In addition, the exploration of the impact of PRDX4 on prostate cell phenotype only includes proliferation and invasion, and further exploration of migration, EMT and so on should be conducted to increase the credibility of the conclusions. Furthermore, this study lacks validation of *in vivo* animal models. Finally, this study did not clarify the underlying mechanisms by which PRDX4 affects the phenotype of prostate cells. In the future, we will further explore and improve the relevant aspects of this study.

In conclusion, we have found that PRDX4, a member of the PRDXs family, has significant diagnostic value for PCa. Moreover, PRDX4 may promote the phenotypic progression of PCa cells, which will open up a new approach for the clinical diagnosis and treatment of PCa.

## Data Availability

The original contributions presented in the study are included in the article/**Supplementary Material**. Further inquiries can be directed to the corresponding authors.

## Supplementary material

Supplementary Table 1 Traditional Chinese medicine targeting prostate cancer with PRDX4.
