# Supplementary material for: Clinical value and molecular role of PRDXs family in prostate cancer
Source: Front Oncol. 2025 Dec 9;15:1713670. doi: 10.3389/fonc.2025.1713670 (PMC12722442; doi:10.3389/fonc.2025.1713670)
Supplement: Supplementary Table 1 — Traditional Chinese medicine targeting prostate cancer with PRDX4. [file Table1.docx]

Supplement Table1 Traditional Chinese Medicine Targeting Prostate Cancer with PRDX4

| Traditional Chinese Medicine | P-Value |
| --- | --- |
| Shi Liu Zi | 0.02006 |
| Yuan Can E | 0.02207 |
| Can Sha | 0.02207 |
| Shi Liu Hua | 0.022365 |
| Shi Liu Pi | 0.022944 |
| Luo Xuan Zao | 0.08645 |
| Ren Shen | 0.089565 |
| Ren Shen Ye | 0.092604 |
| Ren Shen Lu | 0.092668 |
| Ren Shen Hua | 0.092732 |
| Hong Shen | 0.224533 |
| Rou Gui | 0.313748 |
| Gou Qi Zi | 0.358115 |
| Xun Lu Xiang | 1 |
